# A survey study of family members' encounters with healthcare services within the care of older people, psychiatric care, palliative care and diabetes care

**DOI:** 10.1111/scs.13096

**Published:** 2022-07-09

**Authors:** Pardis Momeni, Kristofer Årestedt, Anette Alvariza, Elisabeth Winnberg, Ida Goliath, Åsa Kneck, Janeth Leksell, Mats Ewertzon

**Affiliations:** ^1^ Department of Health Care Sciences Ersta Sköndal Bräcke University College Stockholm Sweden; ^2^ Faculty of Health and Life Sciences Linnaeus University Kalmar Sweden; ^3^ Department of Research Region Kalmar County Kalmar Sweden; ^4^ Capio Palliative Care Dalen Hospital Stockholm Sweden; ^5^ Stockholm Gerontology Research Center Stockholm Sweden; ^6^ Department of Medical Sciences Uppsala University Uppsala Sweden; ^7^ Swedish Family Care Competence Center Kalmar Sweden

**Keywords:** alienation, care context, cross‐sectional, encounter, family members, healthcare services, next of kin, self‐reported

## Abstract

The aim of this study was to describe and compare family members' experiences of approach in encounters with healthcare professionals and possible feelings of alienation in the professional care within four care contexts: the care of older people, psychiatric care, palliative care and diabetes care. The design was an explorative cross‐sectional survey study. Data were collected in Sweden using the Family Involvement and Alienation Questionnaire‐Revised (FIAQ‐R). It measures family members' experiences of the healthcare professionals' approach and the family members' feeling of alienation from the provision of professional care. A total of 1047 questionnaires were distributed to family members using convenient sampling method, of which 294 were included. Data were analysed using rank‐based, non‐parametric statistical methods. The results indicated that most respondents experienced a positive actual approach from the healthcare professionals. Many participants rated the importance of approach at a higher level than their actual experience. Participants in the context of diabetes care reported a more negative actual approach from the healthcare professionals than did participants in the other contexts and considered the healthcare professionals' approach towards them as being less important. The results for the entire group indicated that the participants felt a low level of alienation from the professional care. Participants in the context of the care of older people reported significantly lower level of feeling of being alienated than did participants in the contexts of psychiatric care and diabetes care. The differences between participants in diabetes care and other care contexts can possibly be explained by a more fully implemented self‐care approach among the patients in diabetes care than in the other care contexts. Even though the results are quite positive, it is still important that nurses consider a family‐centred approach to better adapt to the needs of both the family members and the patients.

## BACKGROUND

According to the National Board of Health and Welfare[1], nearly one out of five adults in Sweden helps, cares for or supports a person who is suffering from a long‐term illness or disability. Long‐term illness includes diabetes, some mental illness and conditions included in the care of older people[2]. Today, many people with long‐term illness are in need of palliative care, as the illness often affects physical, psychological, social as well spiritual aspects of life[3]. The patients' long‐term illness can have a wide variety of impacts on family members' life[4]. This is an important area for nursing practice, implicating the need for a family‐focused perspective in the care for people living with long‐term illness. The International Council of Nursing (ICN)[5] points out the importance of nurses acting in partnership with both patients and their family members at all stages of care. Recent research has shown that changes in society regarding the care of persons with long‐term illness have led to shorter periods of hospital care and longer periods of outpatient care. This has resulted in greater responsibility being placed on patients and family members[6, 7, 8]. To manage this responsibility, family members need support and collaboration with the healthcare professionals. Research has pointed to the need for more information and support for family members, and of their greater involvement, in the contexts of the care of older people[9], psychiatric care[10], palliative care[11, 12] and diabetes care[13, 14]. A common feature of these four care contexts are persons suffering from long‐term illnesses or/and disabilities with complex care needs, which results in the family members needing assistance, as well as psychosocial and/or existential support.

The benefits of a family‐focused care are well documented in research, with results highlighting factors such as family participation, support and a partnership approach[15, 16]. However, according to a study by Weimand, Hedelin, Hall‐Lord and Sällström[17], family members of persons with severe mental illness reported a gap between their need of support in order to handle a demanding situation and the support they received from healthcare professionals. Being involved in the decision making about a next of kin has also been highlighted as an important factor for family members. A Swedish study on narrative interviews with families, including persons suffering from long‐term illness, found that the healthcare professionals should work towards including the family members in both discussions and decision making[18]. The study concluded that this is an offer that may not fit every family, but the person with the illness or the family member should at least have the opportunity to choose. Despite these results being confirmed in a study over two decades ago[19], this still seems to be an area of development in both research and in the clinical setting. Recent studies in the context of psychiatric care[20, 21], palliative care[22, 23], care of older people[24, 25] and diabetes care[26] have also focused on the need for more attention to be given to the relationship between nurses and family members. Furthermore, a previous study in the context of psychiatric care found an association between how the family members experience the approach of the healthcare professionals and their feeling of being alienated from the care provided[20]. In that study, the concept alienation refers to feelings of being socially isolated or rejected from the care being provided. Approach characterised by openness, confirmation and cooperation was of great importance for the family members. In nursing research, there has been a paradigm shift during the last decade, which includes a changed view of the nurse's role, with an implementation of a broader definition of person‐centred care that also includes family members. This definition emphasises that changes in one family member, such as those caused by disease and illness, most likely affect all the members of a family with regard to family functioning, beliefs and meaning[27].

Although much is known about family members' experiences of having a close person with long‐term illness, less is known about their experiences of the healthcare professionals' approach towards them and their feelings of alienation regarding the provision of professional care in different care contexts. Such information can be crucial for healthcare authorities and care providers when developing support interventions for family members who have a next of kin with long‐term illness and disabilities.

### Aim and research questions

The aim was to describe and compare family members' experiences of approach in encounters with healthcare professionals and possible feelings of alienation in the professional care within four care contexts: the care of older people, psychiatric care, palliative care and diabetes care.

- How do the family members experience the healthcare professionals' approach and rate its importance?
- Are there any differences between the family members' experiences and what they consider to be important in the healthcare professionals' approach towards them?
- Do the family members experience a feeling of alienation regarding the provision of professional care?
- Are there any differences between the four care contexts regarding the family members' experiences of the healthcare professionals' approach and the family members' feelings of alienation?

## METHOD

### Design

This explorative cross‐sectional survey study is a part of a larger research programme focusing on family member's experience of encounters with healthcare professionals[28]. This study focuses on family members of persons living with long‐term illness in four care contexts. Data collection was carried out from March 2015 until March 2016.

### Participation

The inclusion criterion was that of being an adult family member, aged 18 years or older, of an adult person with long‐term illness within the contexts of the care of older people, psychiatric care, palliative care or diabetes care (type 1). In this study, the term ‘family member’ refers to a relative, partner or friend who currently has contact with a person receiving care within one of the four care contexts. The concept of ‘encounter’ refers to the family member's experience of the healthcare professionals' approach towards them. It includes all the planned and unplanned personal meetings that take place at the healthcare centre between them and the healthcare professionals. It also refers to the family member's feeling of alienation regarding the provision of professional care.

### Data collection and procedure

For data collection, a convenient sampling method was used. A self‐reported questionnaire, the Family Involvement and Alienation Questionnaire‐Revised (FIAQ‐R) was used for data collection. FIAQ‐R has recently been tested for validity and reliability for the target group and includes crucial questions that are of interest to answer the aim of this study.

The heads of care units within the different care contexts were informed by telephone and/or e‐mail to participate in the study. This resulted in 26 care units (19 nursing homes, 2 specialised palliative homecare settings, 2 specialised psychiatric outpatient clinics and 3 hospitals with diabetes units) in the Stockholm area in Sweden. All the care units except 12 nursing homes replied positively. Healthcare professionals or patients at each of the care units identified intended family members to be enrolled in the study. The questionnaires were distributed either by healthcare professionals at the care units (for the care of older people), by patients (for diabetes care) or by the research team who were provided with contact addresses (for the care of older people, palliative care and psychiatric care). All participants received full information regarding the study and the included questionnaire. The questionnaire was distributed to 1047 family members. Attempts were made to increase sample size (within the context of care of older people and palliative care) such as one follow‐up text message and a follow‐up written reminder 1 month after the questionnaires were distributed. The participants that accepted to participate returned the questionnaire via a pre‐stamped envelope to the research leader for this project.

The FIAQ‐R measures family members' experiences of the healthcare professionals' approach (containing the concepts of openness, confirmation, cooperation and continuity) and feeling of alienation from the provision of professional care (containing the concepts of powerlessness and social isolation). The construction of the questionnaire is guided by two theoretical frameworks: the experience of the healthcare professionals' approach by a theory developed by Andershed and Ternestedt[29, 30] and a feeling of alienation by Seeman's[31] conceptualisation of the concept. For further description regarding the theoretical frameworks, see Ewertzon et al [28].

The questionnaire contains 29 items, including the scales of [1]: experience of approach (21 items) and[2] feeling of alienation (8 items). The items in the experience of approach scale are characterised in two steps: (A) by actual experience and (B) by subjective importance. The first of these (A) consists of the family members’ *actual* experience of the professionals' approach and the second (B) describes the *importance* of them. The response categories regarding (A), actual experience, contain four alternatives ranging from ‘completely disagree’[1] to ‘completely agree’[4]. Higher scores indicate a more positive response for the participants' actual experience of the healthcare professionals' approach towards them. The response categories regarding (B), subjective importance, contain four alternatives ranging from ‘of no importance’[1] to ‘of the very highest importance’[4]. A higher score indicates that the participants consider that the healthcare professionals' approach towards them is more important.

The feeling of alienation scale contains four response categories ranging from ‘completely disagree’[1] to ‘completely agree’[4]. A low score indicates a positive response that the participants perceive themselves as not being alienated from the professional care and a high score indicates a negative response, a feeling of alienation. The validity and reliability of the FIAQ‐R have been evaluated in a previous study for the same target group as in this study[28]. The evaluation showed satisfactory psychometric properties in terms of content validity, data quality, homogeneity, factor structure and internal consistency.

In addition, to gain a broader knowledge of the participants' background, demographic characteristics were therefore collected. This included factors such as gender, age, civil status, relationship to the next‐of‐kin (the patient), educational level and place of upbringing.

### Data analysis

Descriptive statistics were used to present socio‐demographic characteristics; to compare the different care contexts, one‐way ANOVA was used continuous and Pearson’s chi‐square test was used for categorical data. The Pearson's chi‐square test was replaced with Fisher’s exact test if the expected frequency in any cell was lower than 5.

As in previous studies using the FIAQ‐R[20, 32, 33], data analysis was performed using rank‐based, non‐parametric statistical methods[34, 35]. To describe the distributions of the response profiles, the median level (Md) and quartiles (Q_1_, Q_3_) were used. A total score of each scale was calculated by the median scoring technique for multi‐items[36].

Kruskal–Wallis test was used to analyse significant comparisons between the four care contexts. The Mann–Whitney *U* test was used as a post hoc test to identify differences between separate care contexts. The Bonferroni–Holm sequential approach was used to identify significant differences between groups using adjusted *p* values.

Percentage agreement (PA) was calculated to evaluate the extent to which the levels of the participants' (A) actual experience and (B) subjective importance agreed. Disagreements between the responses indicated that the participants rated importance at a level that was either higher or lower than the level that they rated experience. The Wilcoxon signed rank test was used to analyse the difference. The overall significance level was set at *p* < 0.05, while the post hoc tests were set at *p* < 0.008, dependent on the numbers of comparisons. Data were analysed using SPSS Statistics 22.1 (IBM Corp.).

### Ethics

The Regional Ethical Review Board in Stockholm has approved the study (No. 2014/583–31). Written informed consent was obtained from the participants, and the participants' responses were kept confidential and coded for the research analyses. The participants’ informed consent included publication of anonymised responses. All participation was voluntary, and participants were informed that they could withdraw if they wished. Funding was provided from the Ersta Sköndal Bräcke University College.

## RESULTS

**FIGURE 1:**
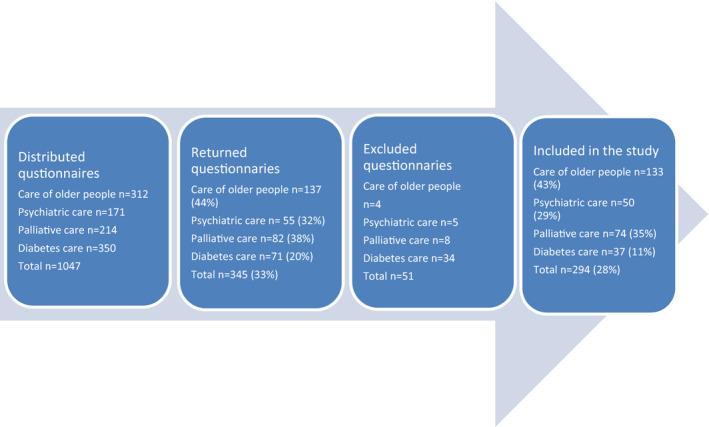
Overview of distributed questionnaires

A total of 1047 questionnaires were distributed and 345 questionnaires were returned, resulting in a total response rate of 33% (see Figure 1). Fifty‐one questionnaires had missing data above 25% of the items in the FIAQ‐R and were therefore excluded. Thus, 294 (28%) completed questionnaires met the eligibility criteria and were included in the statistical analysis in this study.

### Characteristics of the participants

The mean age was 63.0 (SD = 13) years. Most of the participants were females (*n* = 197, 67%), born in Sweden (*n* = 271, 92%), lived with a partner (*n* = 240, 82%) and held a university degree (*n* = 183, 62%). Being an adult child (*n* = 120, 41%) or partner (*n* = 107, 36%) was the most common relationship to the patient. Significant differences between the care contexts were shown for age, civil status and relation to the patient/next of kin and education. Overall, family members in palliative care context were the oldest, most often partner to the patient/next of kin and had lowest education degree (Table 1).

**TABLE 1:** The participants socio‐demographic characteristics for the entire group and different care contexts

Variables	Entire group (*n* = 294) (%)	Care of older people (*n* = 133) (%)	Psychiatric care (*n* = 50) (%)	Palliative care (*n* = 74) (%)	Diabetes care (*n* = 37) (%)	*p*
Gender						
Male	97 (33)	40 (30)	14 (28)	26 (35)	17 (46)	0.257^a^
Female	197 (67)	93 (70)	36 (72)	48 (65)	20 (54)	
Age (years), mean (SD)	63.3 (12.5)	63.0 (11.1)	62.4 (13.0)	67.0 (12.1)	58.1 (15.1)	0.004^b^
Civil status						
Single	53 (18)	28 (21)	17 (34)	4 (5)	4 (11)	<0.001^a^
Partner	240 (82)	104 (78)	33 (66)	70 (95)	33 (89)	
Relation to next of kin						
Parent	41 (14)	0 (0)	28 (56)	3 (4)	10 (27)	<0.001^c^
Sibling	11 (4)	3 (2)	8 (16)	0 (0)	0 (0)	
Husband/wife/co‐habitant	107 (36)	20 (15)	8 (16)	55 (74)	24 (65)	
Child	120 (41)	98 (74)	5 (10)	14 (19)	3 (8)	
Other	14 (5)	12 (9)	1 (2)	1 (1)	0 (0)	
Educational degree						
<Primary degree	3 (1)	1 (1)	0 (0)	2 (3)	0 (0)	0.001^c^
Primary degree	33 (11)	8 (6)	7 (14)	15 (20)	3 (8)	
High school diploma or similar	75 (26)	25 (19)	11 (22)	24 (32)	15 (40)	
University degree or tertiary	183 (62)	99 (74)	32 (64)	33 (45)	19 (51)	
Place of upbringing						
Sweden	271 (92)	125 (94)	45 (90)	66 (89)	35 (95)	0.531^c^
Other Nordic country	9 (3)	2 (1)	1 (2)	4 (5)	2 (5)	
Other European country	3 (1)	1 (1)	1 (2)	1 (1)	0 (0)	
Outside Europe	11 (4)	5 (4)	3 (6)	3 (4)	0 (0)	

^a^ Pearson's chi‐square test.

^b^ One‐way ANOVA.

^c^ Fisher's exact test.

### Experiences of the healthcare professionals´ approach and its importance

Table 2 demonstrates, for the entire group and for different care contexts, the distribution of responses for the experience of approach scale, that is, the participants' actual experience of the healthcare professionals' approach towards them, and for the subjective importance of this, that is, the importance they ascribed to the healthcare professionals' approach towards them. The median level for the entire group for the actual experience of approach was ‘partly agree’[3], which indicated that the participants on average experienced a positive actual approach from the healthcare professionals. The median level of subjective importance was ‘of the very highest importance’[4], which indicated that the participants on average considered the healthcare professionals' approach towards them as being very important. Table 2 also shows significant differences in the distribution of responses among the different care contexts for the participants' experiences of the professional's actual approach towards them (*p* < 0.001) and for the importance they ascribed to this (*p* < 0.001). Significant (*p* < 0.008) differences between the care contexts were found in the post hoc test regarding actual experience of approach in the following contexts: the care of older people and diabetes care, psychiatric care and palliative care, psychiatric care and diabetes care, and palliative care and diabetes care. That is, participants in the context of diabetes care agreed to a lower extent with the statements than did participants in the other care contexts. This indicates that the participants in diabetes care experienced a more negative actual approach from the healthcare professionals than did the participants in the other contexts. Furthermore, in the post hoc test, significant (*p* < 0.008) differences were found in the distribution of responses to subjective importance between the following contexts: the care of older people and diabetes care, psychiatric care and diabetes care, and palliative care and diabetes care. That is, the participants in the context of diabetes care agreed to a lower extent with the statements than did participants in the other contexts.

**TABLE 2:** The distribution of the responses regarding experience of approach scale; experience of actual approach and subjective importance (Md, Q_1_ and Q_3_) for the entire group and different care contexts

	The entire group (*n* = 294) Md (Q_1_;Q_3_)	Care of older people (*n* = 133) Md (Q_1_;Q_3_)	Psychiatric care (*n* = 50) Md (Q_1_;Q_3_)	Palliative care (*n* = 74) Md (Q_1_;Q_3_)	Diabetic care (*n* = 37) Md (Q_1_;Q_3_)	*p* ^a^	Post hoc test^b^
Experience of actual approach^c^	3 (3;4)	3 (3;4)	3 (2;4)	4 (3;4)	2 (1;3)	<0.001	‐‐CDEF
Subjective importance^d^	4 (3;4)	4 (3;4)	4 (3;4)	4 (3;4)	3 (1;3)	<0.001	‐‐C‐EF

^a^ The Kruskal–Wallis test.

^b^ Post hoc test based on Wilcoxon signed rank test. Significant differences (*p* < 0.008) between care context are marked with A = care of older people–psychiatric care, B = care of older people–palliative care, C = care of older people–diabetic care, D = psychiatric care–palliative care, E = psychiatric care–diabetic care, F = palliative care–diabetic care. The letters represent a significant level of *p* < 0.008.

^c^ Response categories: 1 (completely disagree), 2 (partly disagree), 3 (partly agree) and 4 (completely agree).

^d^ Response categories: 1 (of no importance), 2 (of little importance) 3, (of great importance), 4 (of the very highest importance).

### Differences between the experiences of approach and its importance

**FIGURE 2:**
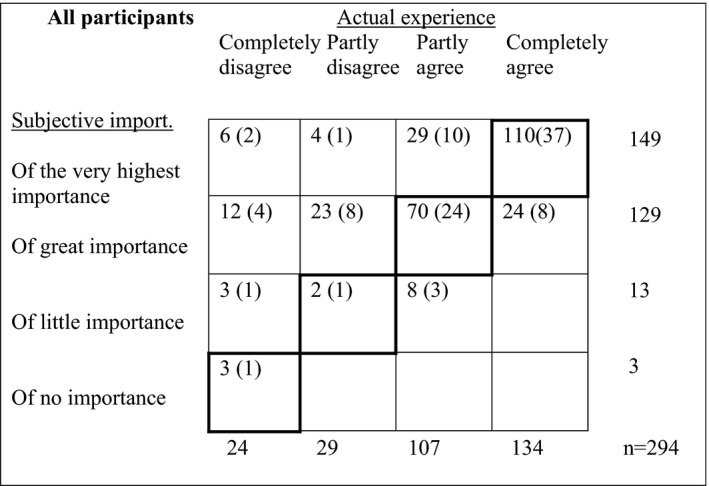
The joint frequency distribution of the assessment by the response categories actual experience and subjective importance. Dark lines indicate agreement. Percentage in brackets

**FIGURE 3:**
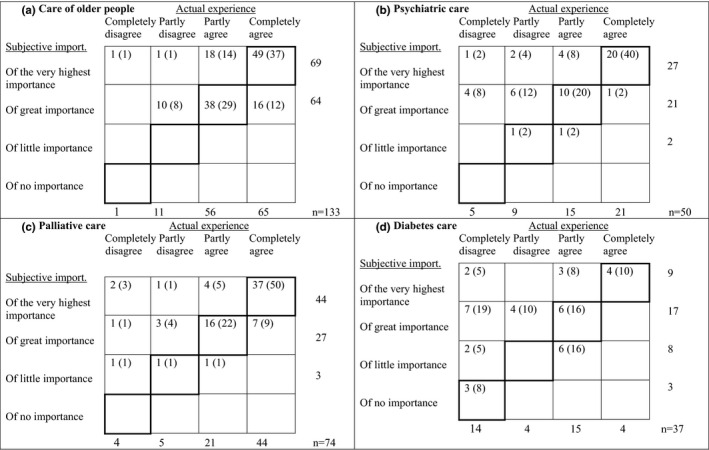
The joint frequency distribution of the assessment by the response categories actual experience and subjective importance with regard to (a) care of older people (PA = 66%), (b) psychiatric care (PA = 62%), (c) palliative care (PA = 73%) and (d) diabetes care (PA = 34%). Dark lines indicate agreement. Percentage in brackets

Figure 2 shows the distributions of the assessments by response category for the actual experience of approach and subjective importance. The percentage agreement (PA) values were 63% and the observations with the greatest level of disagreement were found in the cells above the agreement dialogue, which shows that participants to a greater extent rated subjective importance at a higher level than their actual experience (*p* < 0.001). Figure 3 shows the distributions of assessments by response category for the actual experience of approach and subjective importance for the four care contexts. Except for palliative care, a significant difference in the ratings of actual experience of approach and subjective importance was shown in all care contexts, that is, the care of older people (*p* = 0.026), psychiatric care (*p* = 0.001) and diabetes care (*p* = 0.002).

### Feeling of alienation from the provision of professional care

Table 3 shows the response profile of the feeling of alienation scale for the entire group and for different care contexts, that is, if the participants perceived themselves as being alienated from the provision of professional care. Regarding feeling of alienation, the median agreement level for the entire group was ‘completely disagree’[1], which indicates that, on average, the participants experienced themselves as not being alienated from the professional care. Table 3 also shows significant differences in responses among the care contexts regarding the feeling of being alienated from the professional care (*p* = 0.026). Furthermore, in the post hoc test, significant (*p* < 0.008) differences were found in the distribution of responses to feeling of alienation between the following contexts: care of older people and psychiatric care and care of older people and diabetic care. This indicates that the participants in the context of the care of older people agreed to a lower extent with the items than did participants in the context of psychiatric care.

**TABLE 3:** The distribution of the responses regarding feeling of alienation scale (Md, Q_1_ and Q_3_) for the entire group and different care contexts

	The entire group (*n* = 284) Md (Q_1_;Q_3_)	Care of older people (*n* = 131) Md (Q_1_;Q_3_)	Psychiatric care (*n* = 49) Md (Q_1_;Q_3_)	Palliative care (*n* = 71) Md (Q_1_;Q_3_)	Diabetes care (*n* = 33) Md (Q_1_;Q_3_)	*p* ^a^	Post hoc test^b^
Feeling of alienation^c^	1 (1;3)	1 (1;2)	2 (1;2)	1 (1;2)	2 (1;3)	0.026	A‐C‐‐‐

^a^ The Kruskal–Wallis test.

^b^ Post hoc test; A = care of older people–psychiatric care, B = care of older people–palliative care, C = care of older people–diabetic care, D = psychiatric care–palliative care, E = psychiatric care–diabetic care, F = palliative care–diabetic care. The letters represent a significant level of *p* < 0.008.

^c^ Response categories: 1 (completely disagree), 2 (partly disagree), 3 (partly agree) and 4 (completely agree).

## DISCUSSION

### Discussion of the results

The overall aim of this survey study was to describe and compare family members' experiences of approach in encounters with healthcare professionals and possible feelings of alienation in the professional care in the above‐mentioned context. The results for the entire group indicated that most respondents experienced a positive actual approach from the healthcare professionals. However, many participants rated the importance of approach at a higher level than their actual experience of approach, indicating a discrepancy between their actual experience and what they considered to be important. Furthermore, the results indicated that the participants felt a low level of alienation from the professional care. We also compared the family members' self‐reported encounters with the healthcare services between the four care contexts. Participants in the context of diabetes care reported a more negative actual approach from the healthcare professionals than did participants in the other contexts. However, the participants in diabetes care considered the healthcare professionals' approach towards them as being less important than did participants in the other contexts. Regarding feelings of alienation, the only differences that appeared between the care contexts were among participants in the contexts of the care of older people and the contexts of psychiatric care and diabetes care. Participants in the context of the care of older people experienced a feeling of alienation at a lower level than did participants in those contexts.

To explain the findings, it might be useful to consider the different care traditions regarding family members' encounters with healthcare professionals. Some possible explanations of differences found in our results in the contexts of diabetes care compared to other included care contexts are that people with type 1 diabetes are often encouraged to become more involved in diabetes self‐management. This can possibly be due to self‐management being considered one of the main treatment approaches regarding the disease[37]. Another reason is that in Sweden all people with type 1 diabetes are treated in outpatient care, making it rare for this group of patients to be treated at a ward, meaning that family members are rarely invited to participate in the care meeting in outpatient diabetes care. Furthermore, reviewing the well‐established theories regarding self‐care, for example, in Orem, Taylor and McLaughlin Renpenning[38], they also promote patients being more self‐reliant and responsible for their own care. Although self‐care is crucial for patients living with diabetes, less attention has been given to the family surrounding the patient. It may be that the focus given to self‐management has drawn attention away from the family. In this current study, participants in the context of diabetes care reported a more negative approach, and also considered the healthcare professionals’ approach as being less important compared to the other participants in the included care contexts. This may be explained that the patients suffering from the disease have learned to continuously self‐manage the illness, and may not consider the need for a patient–nurse relationship to be crucial for the well‐being, and in the name of self‐management have been thought to stand by your own and not involve family members in the care process. However, recently published studies regarding ongoing family involvement in diabetes care were shown to be crucial for the transition into a new lifestyle[39, 40, 41]. The question is whether, in the name of self‐management, patients and their families are unconsciously excluded from the healthcare services. This results in a feature scenario where the idea of self‐management will be re‐evaluated so that healthcare professionals involve the patient's social network in the planning of care. Hill, Ward and Gleadle [42] showed that strong social support was vital for ongoing self‐management. When social support was lacking, patients struggled alone with self‐management and felt isolated from the health‐care professionals, indicating that both patients and their family members need support.

In the context of palliative care, our study clearly showed that the participants reported highest levels of actual approach and considered the importance of approach of being the highest level of importance. Also, the feeling of alienation was expressed at a lowest level which indicates positive results for this group. The results can possibly be explained by the fact that there has been considerable research during the last decade regarding the involvement of the family in palliative care, being highly effective and successful in forming care plans and policies [43, 44]. It is also the only care context of the four considered in this study that has incorporated the family into definitions in this area of care, which reflects the progress of published research. So far, the WHO definition has been the most dominant [45], but recently the International Association of Hospice and Palliative Care has suggested a redefinition that includes adopting a broader perspective of the patients [3]. However, family involvement is well established in both of these definitions and in other models for palliative care, described as a necessary and natural part of the given care, both nationally and internationally [46]. In a recommendation for policy regarding Comprehensive and Integrated Palliative Care, family‐focused care was highlighted as important [47].

According to the results, care of older people has great similarities to palliative care, showing overall positive results. At the same time, it differs in some regards especially regarding family members expected involvement in the care. Compared to palliative care units there is less natural space for families in nursing homes, and the quality of available support for families varies considerably from one caring facility to another. A study dealing with the ethical issues of family members in a nursing home setting concluded that there is a need to establish routines in clinical settings for informing and following up family members in a systematic way [25]. In a recent study, family members expressed the need to participate in care meetings and conversations [48, 49]. Other studies confirm these findings, stating the importance of families being able to participate in the care of the older person [8, 50].

Finally, in the group of psychiatric care the results indicated that family members experienced a significantly higher level of alienation than did family members in the context of the care of older people. One explanation may be the form of care and how it is organised, many times being outpatient contact compared to the form of care within nursing home (round‐the‐clock care). Another explanation may be that compulsory care is practiced within psychiatric care, with previous studies showing that family members with experience of compulsory care reported higher levels of alienation than did family members without this experience [51]. It is also possible that younger people with contact within psychiatric outpatient care are more autonomous than elderly people in nursing homes, which may lead to them not wanting family members to be involved in the care [52].

The participants' experience of alienation in this study is in agreement with other studies conducted in the context of psychiatric outpatient care in Sweden [33] and Norway [32].

### Discussion of the method

Several methodological considerations have been undertaken. The low response rate is a threat against the external validity. Therefore, the findings should be generalised with caution.

Another limitation is that no a priori power analysis was conducted for this study. The reason for this is that this study is a part of a larger research programme and that the sample size estimation was based on a psychometric evaluation of the FIAQ‐R [28]. Despite this, the sample size is likely to have been large enough for the analyses that were conducted. Conducting sample size calculations for non‐parametric tests is not possible without knowledge about the distributions. As a rule of thumb for non‐parametric tests, Lehmann and D'Abrera [53] have recommended to calculate the sample size for a corresponding parametric test and add 15% to the required sample size.

The study investigated a group of family members across 14 care units. The design made it possible to obtain a sample that was quite large compared to studies within a specific care unit. We are aware of the limitations regarding the number of family members who received, answered and returned the questionnaire. One‐third of the target group agreed to participate. Low response frequencies are a common problem in questionnaire surveys. Explanations for this in the present study may include the respondents' lack of contact with the healthcare services, language barriers, questionnaire fatigue, exhaustion and burden due to caregiver challenges and deterioration of the patient's condition or patients being deceased.

Additionally, regarding the 350 questionnaires that were distributed by the patients in the context of diabetes care, it is unknown whether they reached the intended respondents, which may explain the low response rate for diabetes care. The sample size and the representativeness of the population could not be determined prior to sending out the questionnaires. When the study was conducted, there were no other sampling procedures known to us for conducting the data collection that would have provided a greater insight into the population. It was therefore decided that the most suitable recruitment strategy was to recruit participants through healthcare professionals or patients at each of the care units. Thus, the low response rate in this group makes it hard to generalise these findings.

The FIAQ‐R has recently been adapted and evaluated in Sweden among the same target group as in this study [28]. This adaptation and evaluation support the suitability of the instrument used in this study. Analysis regarding the total score for missing values showed that 51 questionnaires had missing data above 25% of the items and were therefore excluded. Thirty‐four of those belonged to the context of diabetes care. Of the total excluded questionnaires, 15 were related to factors of approach, 10 to factors regarding alienation and 26 questionnaires concerned both. Thus, there was a consistent distribution of missing data between the scales in the FIAQ‐R.

Besides a low response rate, most of the participants were female, middle‐aged or older, well educated and raised in Sweden. Therefore, the findings should be generalised with care regarding these aspects. Similar observations have been shown in several studies among family members of an adult person with complex care needs (e.g. Ref. [7, 54]).

### Conclusion and clinical implications

The results indicated that most respondents experienced a positive actual approach from the healthcare professionals. However, many participants experience the importance of approach at a higher level than their actual experience. The results for the entire group indicated that the participants felt a low level of alienation from the professional care.

Participants in the context of the care of older people reported significantly lower level of feeling of being alienated than did participants in the contexts of psychiatric care and diabetes care. Even though the results are quite positive, it is still important that nurses consider a broader definition of person‐centred care to better adapt to the needs of both the family members and the person with long‐term illness.

Building on the existing body of research, which has a gap in knowledge regarding family members' self‐reported encounters with healthcare services, this study provides important insights that shed light on the experiences of the family members of persons with long‐term illnesses. More research is needed within this field to better understand the family members surrounding the person with long‐term illness. Qualitative research provides a deeper understanding of family members’ experiences, a key element in not only nursing research but also nursing education. Today's nursing education is mainly oriented towards knowledge regarding care for patients and their diagnoses and symptoms, and less regarding the family surrounding the person. This needs to be further highlighted in both clinical setting and educational. If we fail to include a more family‐focused perspective, we are dealing with increased negative consequences of health for family members that wish to be included. Because it is not a guarantee that if you have family members, they automatically wish to be included. But the choice and the effort from healthcare professionals should be there to ensure that alienation is avoided.

## AUTHOR CONTRIBUTIONS

ME, PM, EW, AA, ÅK and JL was responsible for the conception and design of the study. ME and PM performed the statistical analysis with guidance from KÅ as a statistical advisor. ME and PM drafted the manuscript. All authors helped revise the article critically for intellectual content. All authors contributed to the data collection and agreed on the final version and substantial contributed to the conception and design of the study, analysis and interpretation of the data.

## CONFLICT OF INTEREST

No conflict of interest has been declared by the authors.

## ETHICAL APPROVAL

The Regional Ethical Review Board in Stockholm has approved the study. Written informed consent was obtained from the participants, and the participants’ responses were kept confidential and coded for the research analyses. The participants informed consent included publication of anonymized responses. All participation was voluntary, and participants were informed that they could withdraw if they wished. Funding was provided from Ersta Sköndal Bräcke University College.
